# Modulation of Nociceptive Ion Channels by Protease-Activated Receptor-2 in Inflammatory Pain: Molecular Mechanisms and Therapeutic Potential

**DOI:** 10.3390/ijms27041769

**Published:** 2026-02-12

**Authors:** Haneen Aburamadan, Yosra Lozon, Asha Caroline Cyril, Anagha Nelliyulla Parambath, Najma Mohamed Ali, Reem Kais Jan, Robin Plevin, Rajan Radhakrishnan

**Affiliations:** 1 College of Medicine, Mohammed Bin Rashid University of Medicine and Health Sciences, Dubai P.O. Box 505055, United Arab Emirates; anagha.parambath@dubaihealth.ae (A.N.P.); najma.ali@dubaihealth.ae (N.M.A.); reem.jan@dubaihealth.ae (R.K.J.); rajan.radhakrishnan@dubaihealth.ae (R.R.); 2 Department of Clinical Pharmacy & Pharmacology, Ras Al Khaimah Medical and Health Sciences University, Al Qusaidat, Ras Al Khaimah P.O. Box 11172, United Arab Emirates; 3 Department of Biotechnology, American University of Ras Al Khaimah, Ras Al Khaimah 72603, United Arab Emirates; asha.cyril@aurak.ac.ae; 4 Strathclyde Institute for Biomedical Sciences, University of Strathclyde, 161 Cathedral Street, Glasgow G4 0RE, UK; r.plevin@strath.ac.uk

**Keywords:** ion channels, PAR2, TRPV1, TRPV4, TRPA1, ASIC3, P2X3, Cav3.2, Kv7 (M-current) channels, inflammatory pain

## Abstract

Protease-activated receptor 2 (PAR2) is a G protein-coupled receptor (GPCR) expressed in both the peripheral and central nervous systems. It plays a pivotal role in mediating neuroimmune interactions, particularly in the context of inflammation and pain. Upon activation by proteases, PAR2 modulates nociception through signaling cascades that influence key ion channels, including transient receptor potential (TRP) ion channels vanilloid 1 and 4 (TRPV1 and TRPV4), ankyrin 1 (TRPA1), acid-sensing ion channel 3 (ASIC3), P2X purinoceptor 3 (P2X3), Cav3.2 (T-type Ca^2+^ channel), and potassium Kv7 (M-current) channels, altering their expression and function. Through this crosstalk, PAR2 contributes to heightened neuronal excitability and pain hypersensitivity in various inflammatory conditions. In this narrative review, we highlight and discuss the mechanistic and functional interplay between PAR2 and nociceptive ion channels, which might be contributing to the pathogenesis of inflammatory pain. Targeting these specific molecular interactions between PAR2 and nociceptive ion channels may offer a promising therapeutic strategy for treating inflammatory pain.

## 1. Introduction

Pain, a hallmark of inflammation, is a protective response involving multiple systems. Inflammatory pain refers to the increased sensitivity of pain perception in response to tissue injury that triggers an inflammatory response [1,2]. While acute pain aids in healing, persistent inflammation can lead to chronic pain through increased neural excitability [3,4,5]. Inflammatory pain arises from interactions between the immune system and nervous system [6]. Inflammatory mediators activate nociceptors, receptors expressed in sensory neurons whose cell bodies reside in the dorsal root ganglia (DRG) or trigeminal ganglia (TG), which transmit pain via Aδ and C fibers to the central nervous system (CNS) [5,7]. Chronic exposure to inflammatory mediators lowers neuronal thresholds, leading to peripheral and central sensitization, and resulting in conditions like allodynia and hyperalgesia [8,9].

Peripheral sensitization is driven by mediators such as bradykinin, prostaglandins (e.g., PGE_2_), ATP, and cytokines [3,10,11]. These molecules activate GPCRs and ion channels like transient receptor potential (TRP) ion channels vanilloid 1 and 4 (TRPV1 and TRPV4), ankyrin 1 (TRPA1), acid-sensing ion channel 3 (ASIC3), P2X purinoceptor 3 (P2X3), Cav3.2 (T-type Ca^2+^ channel), and potassium Kv7 (M-current) channels [12,13,14]. Downstream kinases, protein kinase A (PKA), protein kinase C (PKC), and mitogen-activated protein kinases (MAPKs) further amplify this response [15,16,17,18]. Upregulation of ion channels like TRPV1 contributes to chronic pain via hyperalgesic priming [19,20]. Persistent nociceptor input also induces central sensitization, a state of enhanced excitability of CNS neurons [21]. This involves enhanced glutamate and neuropeptide release, reduced GABAergic inhibition, and microglial activation via caspase-6, all contributing to inflammatory hyperalgesia [14,22,23,24].

Neuroimmune interactions further amplify pain by promoting immune cell activation and recruitment. Chemokines like CCL2 (chemokine C-C motif ligand 2) and toll-like receptors (TLRs) signaling in the DRG enhance nociceptor sensitivity and sustain the inflammatory pain state [25,26]. This cascade underlies a wide range of pain in various clinical conditions, including arthritis, endometriosis, inflammatory bowel disease, gout, and diabetic neuropathy [14,27,28,29]. Protease-activated receptor 2 (PAR2), a GPCR, is considered a key modulator in this process and is activated by proteases released during tissue injury and inflammation. Emerging evidence suggests that PAR2 displays functional interactions with several nociceptive ion channels that contribute to amplifying pain signaling. While PAR2 is involved in multiple homeostatic processes, such as epithelial repair and immune function [30], complete antagonism may disrupt these critical roles. Specific non-canonical PAR2-activating proteases were found to selectively mediate PAR2 engagement with ion channels such as TRPV4 driving pain behavior [31]. Complementing this, PAR2 antagonists that selectively block specific downstream signaling pathways while preserving the canonical G-protein signaling (biased antagonist) demonstrated effective analgesia [32]. These findings suggest that targeting specific PAR2-ion channel coupling may provide a more precise and safer therapeutic strategy than global receptor blockade in context of pain management. In contrast, physiological PAR2 functions may engage distinct downstream pathways, allowing for selective inhibition of nociceptive signaling while preserving the essential receptor-mediated homeostatic processes. Importantly, this does not imply direct inhibition of broadly expressed signaling elements; rather, the therapeutic focus is on disrupting localized receptor–channel crosstalk or employing biased antagonists, allosteric modulators, or intracellular inhibitors that selectively block the pain-relevant PAR2 pathways while preserving physiological functions. In this review, we explored the molecular interactions between PAR2 and TRPV1, TRPV4, TRPA1, ASIC3, P2X3, Cav3.2, and Kv7 (M-current) channels and discussed their significance in the development and persistence of inflammatory pain.

## 2. Overview of PAR2 and Its Signaling Pathways

**Figure 1:**
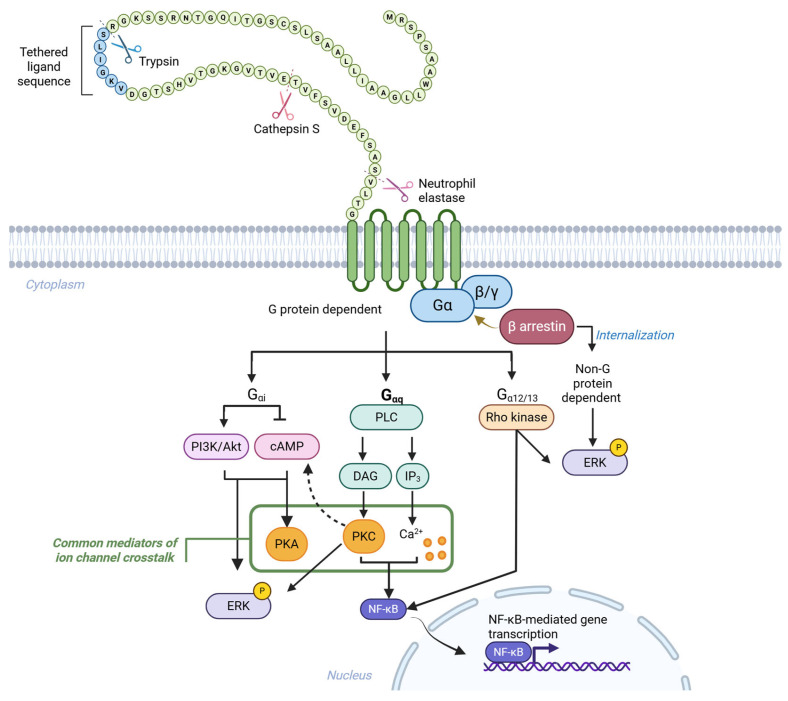
PAR2 activation mechanisms and downstream signaling pathways: PAR2, a GPCR, is activated by proteolytic cleavage of its extracellular N-terminal domain by proteases such as trypsin (canonical cleavage site), cathepsin S, and neutrophil elastase (non-canonical cleavage sites). Cleavage exposes a tethered ligand (blue circles), which activates the receptor. PAR2 signaling involves both G protein- and non-G protein dependent (β-arrestin-dependent) pathways. G protein-dependent pathways involve the activation of G_αq_, inducing PLC activity, leading to the production of DAG and IP_3_. DAG activates PKC, while IP_3_ triggers calcium release from intracellular stores. This leads to activation of the NF-κB (leading to its nuclear translocation and transcriptional activation) and ERK pathways. PKC can also stimulate specific adenylyl cyclase isoforms (shown as a dotted arrow), leading to cAMP generation and PKA activation. G_αi_ activation inhibits (blunt arrow) adenylyl cyclase, reducing cAMP levels and activating PI3K/Akt, which contribute to activation of the ERK signal. G_α12/13_ activation stimulates Rho GTPases, leading to Rho kinase activation and contributing to cytoskeletal reorganization and ERK/NF-κB activation. Non-G protein-dependent pathways: β-arrestins facilitate receptor internalization via clathrin-coated pits and serve as scaffolds for non-G protein-dependent signaling cascades involving, predominantly, ERK activation. This pathway provides sustained signaling distinct from transient G protein-mediated responses and regulates receptor recycling and trafficking. Abbreviations: PAR2, protease-activated receptor 2; PLC, phospholipase C; DAG, diacylglycerol; IP_3_, inositol trisphosphate; PKC, protein kinase C; NF-κB, nuclear factor kappa B; ERK, extracellular signal-regulated kinase; P, phosphorylation; cAMP, cyclic adenosine monophosphate; PI3K, phosphoinositide 3-kinase. Solid arrows (→): activation; blunt arrow (⟞): inhibition; dotted arrow (--->): isoform-specific interaction; color coding: green = PAR2; blue = G proteins; orange = kinases/Ca^2+^ ions (PKC, PKA) involved in ion channels crosstalk; red = β-arrestin; purple = transcriptional regulators (ERK, NF-κB).

Like other GPCRs, the PAR2 signaling is activated via direct interactions with heterotrimeric G proteins, composed of Gα, Gβ and Gγ subunits [33]. PAR2 is activated when specific proteases cleave its extracellular N-terminal domain, exposing a tethered ligand (a short amino acid sequence) that binds intramolecularly to initiate receptor signaling. Trypsin cleaves a “canonical” site (R36↓S37), triggering the canonical G protein-dependent and -independent signaling [34] (Figure 1). Short synthetic peptides that mimic the TL sequence (SLIGKV-NH_2_ in humans and SLIGRL-NH_2_ in rodents) can activate PAR2 independently of proteolytic cleavage [35]. Upon activation, PAR2 couples primarily to G_αq/11_, G_αi/o_, and G_α12/13_ [36], initiating downstream signaling cascades that include Ca^2+^ mobilization, extracellular signal-regulated kinases 1 and 2 (ERK1 and 2) phosphorylation, Rho kinase activation, protein kinase B (Akt) signaling, and modulation of cyclic adenosine monophosphate (cAMP) levels [37]. PAR2/Gαq/11/Ca^2+^/protein kinase C (PKC) pathway ultimately activates nuclear factor-κB (NF-κB), promoting transcription of proinflammatory cytokines and adhesion molecules such as intercellular adhesion molecule-1 (ICAM-1) [38]. In parallel, β-arrestin acts as a scaffold for G protein-independent signaling, facilitating receptor internalization and sustained ERK activation via the mitogen-activated protein kinase (MAPK) pathway [39,40]. Internalized PAR2 is trafficked through endosomes, where it is either targeted for lysosomal degradation or recycled to the plasma membrane through Golgi-derived stores [41,42]. This dynamic regulation enables receptor resensitization and fine-tuning of inflammatory signaling [43].

In addition to canonical activation, PAR2 can be cleaved at alternative, “non-canonical” sites by various proteases, initiating biased signaling; a phenomenon in which different ligands selectively engage distinct intracellular pathways (Figure 1). This functional selectivity enables PAR2 to mediate diverse, context-specific cellular responses [44]. For example, neutrophil elastase activates ERK1/2, without inducing Ca^2+^ mobilization or β-arrestin recruitment [12,45]. Interestingly, the synthetic peptide corresponding to the tethered ligand revealed by elastase fails to generate PAR2 activity. In contrast, cathepsin-S cleaves PAR2 at a site distant from trypsin and elicits a cAMP response without Ca^2+^ mobilization or ERK signal or β-arrestin recruitment, while its synthetic peptide mimicking the cathepsin S-revealed sequence retains partial agonistic activity [31,46]. These differences are thought to arise from protease-specific conformational changes in PAR2, highlighting the receptor’s structural plasticity [29,34].

Emerging evidence within GPCR research indicated that pain-related outcomes are linked to ligand-dependent stabilization of specific receptor conformations. These conformations were found to bias coupling to specific intracellular signaling transducers. Recent structural and pharmacological studies, particularly utilizing cryo-electron microscopy, have demonstrated that GPCRs exhibit specific conformations that preferentially couple to G-protein (canonical) or β-arrestin (non-canonical) pathways, leading to different functional outcomes in the context of pain [47]. Given that PAR2 agonists and antagonists demonstrated biased signaling profiles, this mechanistic concept needs to be considered when evaluating PAR2-mediated regulation of ion channel sensitization in diseases. Understanding the structural requirements that govern PAR2-biased signaling will be essential for defining its analgesic potential and for the rational development of PAR2 antagonists or modulators that selectively disrupt the pathological ion channels coupling to PAR2, while preserving physiological signaling.

## 3. PAR2-Driven Sensitization Mechanisms in Inflammatory Pain

PAR2 has gained special attention for its role in inflammation, including inflammatory pain. Activated by proteases such as trypsin and mast cell tryptase during tissue injury, PAR2 has been found to be evident in inflammatory responses in various tissues [48].

### 3.1. Signaling Mechanisms Driving Pain Sensitization

Upon activation, PAR2 stimulates multiple intracellular signaling cascades that contribute to neural sensitization. The canonical Gq leads to increased intracellular Ca^2+^ via IP_3_ and activation of protein kinase C (PKC) through DAG. These signaling events are required for PAR2-mediated inflammatory sensitization of nociceptive neurons [49,50]. In addition, PAR2 activation stimulates MAPK signaling, including ERK1/2 and p38, which are particularly central to both peripheral and central sensitization [15]. The inhibition of MAPK cascade attenuated neutrophil elastase-induced hyperalgesia to levels observed in PAR2-knockout rodents, suggesting its critical role [51]. β-arrestin-dependent pathways also contribute to pain via sustained ERK activation, and a biased PAR2 antagonist targeting this signaling arm has shown efficacy in reducing spontaneous and evoked nociceptive behaviors in vivo [32]. In addition, PAR2-mediated NF-κB activation leads to enhanced release of proinflammatory cytokines, that in turn sensitize peripheral nociceptors and contribute to sustained inflammatory pain [52,53,54]. The effects of the released proteases and cytokines create a feedback loop that intensifies and maintains pain sensation.

### 3.2. Peripheral and Central Sensitization via PAR2

PAR2 is expressed in mammalian peripheral neural tissue, including afferent sensory neuron terminals and cell bodies [55], in addition to mesenteric and submucosal neurons [56], positioning it to directly influence nociceptive transmission. Although PAR2 is expressed in DRG neurons associated with itchiness, evidence showed that these neurons contribute to pain sensitization. For example, Liu et al. demonstrated that PAR2 activation with selective agonist peptide (SLIGRL) potentiated TRPV1 responses in DRG cultures and induced thermal hyperalgesia in vivo, effects that were abolished in PAR2-deficient mice [56]. Interestingly, trypsin-induced itch was paradoxically enhanced, suggesting a minimal role of PAR2 in itchiness. Complementing this, Hasseler et al. showed that PAR2 is expressed in a small subset (~3–4%) of DRG nociceptors that co-express TRPV1 and markers for itch neurons. The PAR2 agonist produced robust mechanical allodynia, facial grimacing that was abolished in sensory neuron-specific PAR2-knockouts [57]. Experimental models demonstrate that peripheral PAR2 activation induces both enhanced mechanical allodynia [45] and thermal hyperalgesia in rodents [56,58] whether induced by proteases or synthetic PAR2-activating peptides (PAR2-AP). Central PAR2 activation also contributes to nociceptive sensitization. Intrathecal administration of PAR2-AP enhances thermal hyperalgesia in a peripheral cutaneous inflammation model [59]. Supporting these behavioral results, in vitro electrophysiological recordings showed increased frequency and amplitude of dorsal horn neuronal activity following PAR2-AP application to ex vivo spinal cord slices [57] These nociceptive effects are tightly linked to PAR2-mediated modulation of ion channels, particularly members of the transient receptor potential (TRP) family, including TRPV1 and TRPV4 [37].

## 4. Role of PAR2 in Modulation of Ion Channels

**Figure 2:**
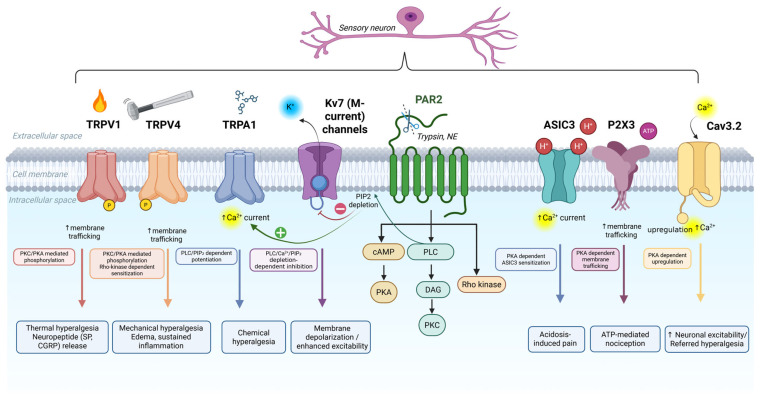
Crosstalk between PAR2 and ion channels in sensory neurons mediating inflammatory pain. Schematic illustration of the molecular interplay between PAR2 and key ion channels involved in nociceptive signaling in sensory neurons: TRPV1, TRPV4, TRPA1, ASIC3, P2X3, Cav3.2, and Kv7 (M-current) channels. Activation of PAR2 by proteases (e.g., trypsin, neutrophil elastase) triggers multiple intracellular signaling pathways, cAMP/PKA, PLC/DAG/PKC, and Rho kinase, which modulate the function of these ion channels. TRPV1 and TRPV4: Sensitized via PKC- and PKA-mediated phosphorylation and membrane trafficking, leading to thermal and mechanical hyperalgesia, and enhanced neuropeptide (substance P, calcitonin gene-related peptide CGRP) release (TRPV1). TRPA1: Activated downstream of PLC-mediated PIP_2_ depletion, resulting in increased Ca^2+^ currents (green + circle) and chemical hyperalgesia. ASIC3: Potentiated through PKC and PKA activation, amplifying proton-gated Ca^2+^ currents and leading to acidosis-induced pain. P2X3: Modulated via PKC/PKA-dependent trafficking, increasing surface expression and facilitating ATP-mediated nociception. Voltage-gated ion channels (Cav3.2 and Kv7) represent voltage-gated calcium and potassium channels, respectively. Cav3.2 is upregulated via PKA signaling leading to increased excitability and referred hyperalgesia, while Kv7 is inhibited through PLC–Ca^2+^–PIP_2_ depletion (red − circle), resulting in neural depolarization and increased excitability. Solid arrows (→): activation; blunt arrow (⟞): inhibition; color coding: TRPV1 = red, TRPV4 = orange, TRPA1 = blue, Kv7 channels = purple, ASIC3 = turquoise, P2X3 = light purple, Cav3.2 = yellow; blue boxes at lower side of the figure indicate functional outcomes of PAR2-mediated modulation of each ion channel.

**Figure 3:**
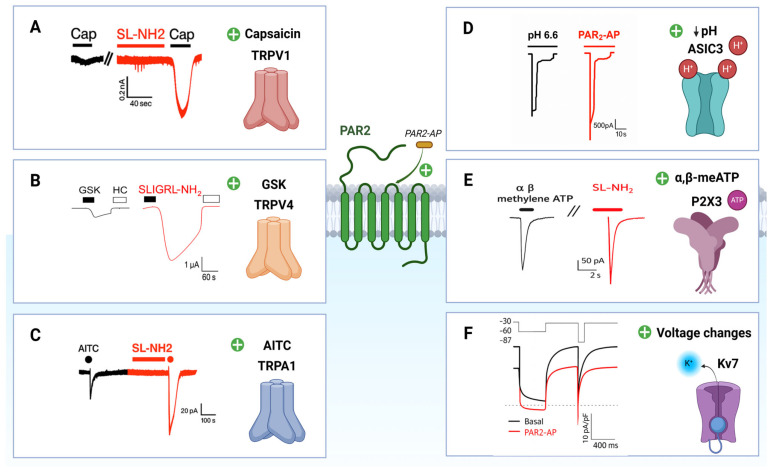
Representative electrophysiological traces showing PAR2-mediated modulation of ion channel activity involved in inflammatory pain. PAR2 activation by specific agonists (e.g., SL-NH_2_ or PAR2-AP) enhances neuronal currents through TRPV1 (**A**), TRPV4 (**B**), TRPA1 (**C**), ASIC3 (**D**), and P2X3 (**E**), while inhibiting the M-current carried by Kv7 channels (**F**). Green plus signs show the specific ion channel agonist/activating condition. Representative traces are redrawn from published studies ((**A**): panel A, Figure 4 of [60]; (**B**): panel A, Figure 5 of [61]; (**C**): panel B, Figure 5 of [62]; (**D**): panel A, Figure 5 of [63]; (**E**): panel B, Figure 1 of [64]; (**F**): panel A, Figure 1 of [48]). Black traces = baseline; red traces = after PAR2 activation. Increased currents contribute to hyperexcitability and inflammatory pain, whereas Kv7 inhibition promotes depolarization.

A total of 27 papers were selected according to the eligibility criteria and are summarized in Table 1. These studies provide compelling evidence for the modulatory role of PAR2 on various nociceptive ion channels involved in inflammatory pain. A graphical summary illustrating the key mechanisms and signaling pathways through which PAR2 interacts with these ion channels is presented in Figure 2. Representative electrophysiological traces illustrating the effect of PAR2 activation on ion channel currents are depicted in Figure 3.

**Table 1:** Summary of PAR2 interactions with ion channels in inflammatory pain models.

Ion Channel	Key Experimental Models/Techniques	Mechanistic Summary of Findings	Functional Significance	Citation
TRPV1	Mouse model of inflammatory pain; HEK293, DRG neurons; Ca^2+^ imaging; electrophysiology; Western blot (TRPV1 phosphorylation) and IP; behavioral assays (thermal hyperalgesia).	PAR2 activation (SLIGRL-NH_2_/trypsin/tryptase) enhanced TRPV1 currents, Ca^2+^ influx, and neuropeptide (SP/CGRP) release via PLC/PKC, and increased TRPV1 phosphorylation. Effect reversed by TRPV1 antagonist or knockout.	PAR2–TRPV1 coupling in thermal hyperalgesia.	Amadesi et al. (2004) [65]
	Mouse model of inflammatory pain; HEK293, DRG neurons; electrophysiology; immunohistochemistry (Fos); behavioral assays (thermal and mechanical hypersensitivity).	PAR2 activation (SL-NH_2_/trypsin/tryptase) potentiated TRPV1 responses to capsaicin, heat, and protons via PKC signaling. PAR2-induced enhanced Fos expression and pain behaviors were reduced in TRPV1-deficient mice.	PAR2–TRPV1 sensitization is PKC-dependent underlying thermal and mechanical hyperalgesia.	Dai et al. (2004) [60]
	Mouse model of inflammatory pain; HEK293, DRG neurons; electrophysiology; Ca^2+^ imaging; behavioral assays (thermal hyperalgesia).	PAR2 activation (SLIGRL-NH_2_/trypsin) sensitized TRPV1 via PKCε and PKA, increasing TRPV1 currents and Ca^2+^ influx. Co-application with capsaicin induced robust thermal hyperalgesia in vivo.	PAR2–TRPV1 sensitization is PKCε/PKA-dependent contributing to inflammatory thermal hyperalgesia.	Amadesi et al. (2006) [66]
	Rat and mouse arthritis models; behavioral assays (mechanical allodynia, weight bearing); ELISA (IL-1β, TNF-α)	Intra-articular PAR2 activation (SLIGRL-NH_2_) induced secondary mechanical hyperalgesia and spontaneous pain, which were attenuated by TRPV1 antagonism or genetic deletion. PAR2-induced IL-1β release was unaffected by TRPV1 inhibition.	TRPV1 mediates PAR2-induced mechanical hypersensitivity in arthritis, independent of cytokine release.	Helyes et al. (2010) [67]
	Rat arthritis model; electrophysiology (joint afferent recordings); intravital microscopy (leukocyte kinetics).	PAR2 activation (2-fu-LIGRLO-NH_2_) increased joint afferent firing and promoted leukocyte rolling and adhesion. These effects were blocked by TRPV1 and NK1 receptor antagonists.	PAR2–TRPV1–NK1 axis contributes to peripheral sensitization and inflammation in arthritis.	Russell et al. (2012) [68]
	Rat model of inflammatory pain; electrophysiology; behavioral assays (thermal and mechanical hypersensitivity).	Intrathecal PAR2 activation (SLIGKV-NH_2_) increased synaptic transmission and thermal hyperalgesia via TRPV1-dependent mechanisms in the spinal dorsal horn.	Spinal PAR2–TRPV1 signaling enhances nociceptive transmission and thermal sensitivity.	Mrozkova et al. (2016) [69]
	Mouse model of myocardial ischemia/reperfusion; Langendorff heart preparation; hemodynamic analysis; TTC staining.	PAR2 activation (SLIGRL) reduced infarct size and improved cardiac function through TRPV1 sensitization via a 12-LOX-dependent pathway, promoting CGRP and SP release. Effects were abolished in TRPV1-deficient mice.	TRPV1 mediates PAR2-driven cardioprotection through neuropeptide-dependent signaling.	Zhong et al. (2019) [70]
	Rat model of carrageenan-induced inflammatory pain; electrophysiology; behavioral assay (thermal hyperalgesia).	Intrathecal PAR2 inhibition (FSLLRY-NH_2_) reduced thermal hyperalgesia and attenuated excitatory synaptic transmission in the dorsal horn via TRPV1 and kinase-dependent pathways.	PAR2–TRPV1 signaling sustains spinal sensitization and thermal pain in inflammatory states.	Mrozkova et al. (2021) [59]
TRPV1, Cav3.2 (T-type Ca^2+^ channel)	Mouse model of cystitis; human bladder epithelial T24 cells; behavioral assay (referred hyperalgesia).	PAR2 activation (SLIGRL) increased COX-2–mediated PGE_2_ production, leading to TRPV1 sensitization and Cav3.2 upregulation via PKA signaling. TRPV1 blockade prevented referred hyperalgesia.	PAR2–TRPV1 signaling promotes visceral hypersensitivity through prostanoid–PKA pathways.	Tsubota et al. (2018) [71]
TRPV4	Mouse model of inflammatory pain; HBE cells, HEK293, DRG neurons; Ca^2+^ imaging; electrophysiology; neuropeptide assays; behavioral assay (mechanical hyperalgesia).	PAR2 activation (SLIGRL-NH_2_) sensitized TRPV4 by enhancing Ca^2+^ influx and currents via PLCβ, PKC, and PKA pathways. This increased SP and CGRP release from dorsal horn neurons.	PAR2–TRPV4 signaling amplifies mechanical hypersensitivity through kinase-dependent sensitization and neuropeptide release.	Grant et al. (2007) [72]
	Rat model of inflammatory pain; HEK293 cells, DRG neurons; Ca^2+^ imaging; inflammation assessment (paw thickness).	PAR2 activation (trypsin, SLIGRL-NH_2_) induced sustained TRPV4-mediated Ca^2+^ influx via arachidonic acid derivatives (e.g., 5,6-EET) and Src-dependent Tyr-110 phosphorylation. TRPV4 deficiency reduced paw edema.	TRPV4 activation by PAR2 contributes to sustained inflammatory signaling via lipid mediators and tyrosine phosphorylation.	Poole et al. (2013) [73]
	Mouse model of inflammatory pain; HEK293, KNRK cells, DRG neurons, Xenopus oocytes; Ca^2+^ imaging; electrophysiology; behavioral assays (mechanical hyperalgesia, paw edema)	Cathepsin S cleaved PAR2 at a non-canonical site (E^56^↓T^57^), inducing biased Gαs/cAMP/PKA signaling without Ca^2+^ mobilization or ERK activation. This sensitized TRPV4 and enhanced neuronal excitability and mechanical hyperalgesia.	Biased PAR2 signaling via Cathepsin S drives TRPV4-dependent mechanical pain and inflammation.	Zhao et al. (2014) [30]
	Mouse model of inflammatory pain; HEK293 cells; Ca^2+^ imaging; behavioral assay (mechanical hyperalgesia)	PAR2 activation (SLIGRL-NH_2_, trypsin) induced sustained TRPV4-mediated Ca^2+^ influx, which was blocked by the tyrosine kinase inhibitor bafetinib. Bafetinib also attenuated PAR2–TRPV4-induced mechanical hyperalgesia in vivo.	Tyrosine kinase–dependent TRPV4 sensitization underlies PAR2-mediated mechanical hypersensitivity.	Grace et al. (2014) [74]
	Xenopus oocytes; electrophysiology; intracellular Ca^2+^ chelation (BAPTA-AM)	PAR2 activation (trypsin, SLIGRL-NH_2_) enhanced TRPV4 responses independent of intracellular Ca^2+^ signaling. This sensitization was independent of intracellular Ca^2+^ signaling (BAPTA-AM had no effect). Neutrophil elastase, a biased PAR2 agonist, sensitized TRPV4 via the Rho-kinase pathway.	TRPV4 sensitization by biased PAR2 agonists occurs through Ca^2+^-independent, Rho-kinase–mediated mechanisms.	Sostegni et al. (2015) [61]
	Mouse model of inflammatory pain; KNRK cells, HEK293 cells, Xenopus oocytes; electrophysiology; Ca^2+^ imaging; behavioral assays (mechanical hyperalgesia, paw edema)	Neutrophil elastase, a biased PAR2 agonist, selectively activated Gαs/cAMP/PKA signaling (not Gαq/Ca^2+^), leading to TRPV4 sensitization, ERK phosphorylation, and TRPV4-dependent mechanical hyperalgesia with inflammatory edema.	Biased PAR2 signaling promotes TRPV4-dependent pain and inflammation via Gαs–PKA pathways.	Zhao et al. (2015) [45]
	Mouse model of LPS-induced acute lung injury; alveolar macrophage culture; cytosolic Ca^2+^ measurement	PAR2 activation (thrombin) increased cAMP in alveolar macrophages, which suppressed TRPV4-mediated Ca^2+^ influx and downstream NFAT activation. PAR2 deletion enhanced TRPV4 activity and inflammation; restoration of PAR2 or TRPV4 inhibition reversed the effect.	PAR2 negatively regulates TRPV4 in lung inflammation, contributing to resolution of acute lung injury.	Rayees et al. (2019) [75]
TRPA1	Rat model of inflammatory pain; HEK293 cells, DRG neurons; electrophysiology; behavioral assay (chemical-induced acute pain)	PAR2 activation (SLIGRL-NH_2_) enhanced TRPA1 currents in vitro through a PLC–PIP_2_–dependent pathway. In vivo, PAR2 agonist increased nocifensive responses to TRPA1 agonists.	PAR2–TRPA1 coupling heightens chemical nociception via PLC–PIP_2_ signaling.	Dai et al. (2007) [62]
	Rat model of cystitis; cystometry; Western blot (TRPA1 expression); behavioral assay (mechanical hypersensitivity)	Inhibition of PAR2 (FSLLRY-NH_2_) reduced TRPA1 upregulation in spinal tissue and alleviated bladder hyperactivity and mechanical pain in inflamed rats.	Spinal PAR2–TRPA1 signaling contributes to cystitis-associated pain and bladder dysfunction.	Chen et al. (2016) [76]
	Multiple mouse models of migraine; behavioral assay (cutaneous allodynia)	Cutaneous allodynia induced by TRPA1 activation was abolished by PAR2 inhibition using the monoclonal antibody MEDI0618.	PAR2 signaling maintains TRPA1-driven allodynia in migraine, indicating a therapeutic target.	Kopruszinski et al. (2025) [77]
TRPA1, TRPV1	Mouse model of pancreatitis; immunohistochemistry (spinal Fos expression); behavioral assay (referred hyperalgesia)	PAR2 activation (SLIGRL-NH_2_) in the pancreas increased spinal Fos expression. TRPA1 inhibition blocked this effect; TRPV1 inhibition reversed referred hyperalgesia, which was further reduced by combined TRPA1 and TRPV1 blockade.	TRPA1 and TRPV1 act synergistically downstream of PAR2 to mediate pancreatitis-associated referred pain.	Terada et al. (2013) [78]
TRPV1, TRPA1, TRPV4	Rat model of oral mucositis; behavioral assays (spontaneous pain, mechanical allodynia)	PAR2 activation by neutrophil elastase sensitized TRPV1 and TRPA1 to mediate spontaneous pain, and TRPV4 to drive prolonged mechanical allodynia.	PAR2–TRPV1/TRPA1 signaling underlies spontaneous pain, while PAR2–TRPV4 drives mechanical allodynia in mucositis.	Ito et al. (2017) [79]
ASIC3, TRPV1	Primary human esophageal epithelial cells (HEECs); ATP bioluminescence assay; siRNA knockdown; Western blot; IP	PAR2 activation (trypsin, SLIGKV-NH_2_) enhanced weak acid–induced ATP release via TRPV1 and ASIC3 sensitization. TRPV1, but not ASIC3, was phosphorylated. Effects were reduced by antagonists or gene silencing.	PAR2 enhances acid-induced ATP signaling in esophageal cells via TRPV1 and ASIC3, contributing to sensory hypersensitivity.	Wu et al. (2015) [80]
ASIC3	Rat model of acidosis-induced inflammatory pain; CHO cells, DRG neurons; electrophysiology; behavioral assay (acetic acid test)	PAR2 activation (2-fu-LIGRLO-NH_2_) potentiated ASIC3 currents via PLC, PKC, and PKA signaling. This enhanced nocifensive behavior in response to tissue acidosis.	PAR2–ASIC3 signaling amplifies acid-evoked pain through kinase-dependent sensitization.	Wu et al. (2017) [63]
P2X3	Rat model of α,β-meATP–induced acute pain; DRG neurons; immunohistochemistry (c-Fos); behavioral assay (chemical nociception)	PAR2 activation (SLIGRL-NH_2_) enhanced α,β-meATP–evoked nocifensive behavior and increased spinal c-Fos expression, indicating elevated P2X3 activity and central sensitization.	PAR2 enhances P2X3-mediated pain signaling and spinal activation during acute chemical nociception.	Zhu et al. (2006) [81]
	DRG neurons; electrophysiology	PAR2 activation (SL-NH_2_ or trypsin) reduced the amplitude but accelerated the opening kinetics of P2X3 currents, indicating a dual modulatory effect on channel function.	PAR2 modulates P2X3 gating properties, altering sensory neuron excitability.	Lu et al. (2010) [82]
	Rat model of α,β-meATP–induced pain; DRG neurons; electrophysiology; Western blot; immunoprecipitation (IP); immunohistochemistry (ERK); P2X3 trafficking assays; behavioral assay (chemical nociception test)	PAR2 activation (SL-NH_2_ or trypsin) enhanced P2X3 currents via PKA/PKC-dependent membrane trafficking. This increased ERK phosphorylation and nocifensive behavior in response to α,β-meATP.	PAR2 enhances P2X3-mediated nociception by promoting receptor trafficking and spinal signal amplification.	Wang et al. (2012) [64]
Kv7 (M-current potassium channel)	Rat model of inflammatory pain; DRG neurons; electrophysiology; behavioral assays (thermal and mechanical hyperalgesia)	PAR2 activation (2f-LIGRLO-amide, trypsin) inhibited Kv7 (M-current) via a PLC-dependent pathway involving intracellular Ca^2+^ increase and PIP_2_ depletion, leading to neuronal depolarization and excitability. Co-application with Kv7 blocker (XE991) enhanced nociception.	PAR2 suppresses Kv7 activity, increasing sensory neuron excitability and contributing to inflammatory hyperalgesia.	Linley et al. (2008) [48]

Abbreviations: DRG (dorsal root ganglion), EET (Epoxyeicosatrienoic acid), HEK (human embryonic kidney), HBE (human bronchial epithelial), PKA (protein kinase A), PKC (protein kinase C), PLC (phospholipase C), PIP_2_ (phosphatidylinositol 4,5-bisphosphate), ERK (extracellular signal-regulated kinase), SP (substance P), CGRP (calcitonin gene-related peptide), COX-2 (cyclooxygenase-2), PGE_2_ (prostaglandin E_2_), FSLLRY-NH_2_ (a PAR2 antagonist peptide), SLIGRL-NH_2_/SL-NH_2_ (PAR2 agonist peptides), TTC staining (triphenyl tetrazolium chloride staining), T24 cells (human bladder epithelial carcinoma cell line), CHO cells (Chinese hamster ovary cells), Fos (an immediate early gene product used as a marker of neuronal activation), NFAT (nuclear factor of activated T cells), LOX (lipoxygenase), NK1 (neurokinin 1 receptor), BAPTA-AM (1,2-Bis-(o-Aminophenoxy)-ethane-N,N,N′,N′-tetraacetic acid, tetraacetoxymethyl ester: a membrane-permeable calcium chelator), IP (Immunoprecipitation).

### 4.1. TRPV1

The functional interaction between PAR2 and TRPV1 is supported by a total of twelve in vivo and in vitro studies across multiple experimental systems. TRPV1 is one of the six members of the cationic transient receptor potential vanilloid (TRPV) ion channels. It is highly expressed in primary sensory neurons and in a subset of DRG and trigeminal neurons. It serves as a molecular sensor for thermal and chemical noxious stimuli and is activated by capsaicin (the active ingredient of chili peppers), noxious heat (>42 °C), toxins, and protons [83]. Immunofluorescence studies have demonstrated co-localization of PAR2 and TRPV1 in DRG neurons, where most capsaicin-responsive neurons also responded to PAR2-AP [65]. In vitro, PAR2 activation leads to a transient rise in intracellular Ca^2+^ concentration, potentiating capsaicin-evoked TRPV1 currents (Figure 3A), and lowers thermal activation thresholds of TRPV1 [60,66]. These molecular findings are further supported by behavioral studies. In models of peripheral inflammation, treatment with a TRPV1 antagonist significantly reduced PAR2-AP-induced thermal hyperalgesia [65], while TRPV1 genetic deletion abolished these effects completely [60,65]. Examining central PAR2 activation, intrathecal administration of PAR2-AP elicited thermal hypersensitivity that was blocked by spinal TRPV1 inhibition [59,69], and was absent in PAR2-deficient mice [84]. Mechanistically, PAR2-induced TRPV1 sensitization is mediated through PLCβ, PKCε, and PKA, where pharmacological inhibition of these intracellular enzymes suppressed TRPV1-evoked Ca^2+^ signals in culture and attenuated thermal hypersensitivity in vivo [66].

### 4.2. TRPV4

For TRPV4, a total of eight studies provided evidence of functional interaction between PAR2 and this channel utilizing various models of inflammatory pain. TRPV4 is another member of the TRPV ion channels, activated by lipid mediators, as well as osmotic and mechanical stress. Hence, it plays a major role in osmoregulation and mechanotransduction in the body [73,83]. TRPV4 and PAR2 are co-expressed in primary sensory neurons and in a subset of DRG neurons, and their coupling is significant for the induction of inflammatory hyperalgesia [72]. In cultured DRG neurons and transfected HEK cells, PAR2 activation potentiated TRPV4-mediated calcium influx and enhanced responses to the TRPV4 agonist 4α-Phorbol 12,13-didecanoate (4αPDD). This sensitization was dependent on PLCβ and multiple kinase pathways, including PKA, PKC, and PKD, as evidenced by pharmacological inhibition studies [72]. This sensitization is mediated by PLCβ-, PKA-, PKC-, and PKD-dependent signaling, as shown by the use of specific kinase inhibitors. In the same study, intraplantar injection of PAR2 agonist induced mechanical hyperalgesia in mice and enhanced pain responses of TRPV4 agonist (4αPDD). Further mechanistic studies have shown that sustained PAR2 signaling leads to increased intracellular Ca^2+^ in a TRPV4-dependent manner, and that both pharmacological blockade and genetic deletion of TRPV4 abolish this response and attenuate PAR2-induced inflammation [73]. Inhibitors of both phospholipase A2 and cytochrome P450 epoxygenase required for catalysis of arachidonic acid markedly attenuated the sustained response of PAR2, suggesting that PAR2 activation leads to increased endogenous release of arachidonic acid derivatives such as 5,6-Epoxyeicosatrienoic acid which ultimately activates TRPV4 [73]. The activation of TRPV4 by PAR2 also requires phosphorylation at a key tyrosine residue (Tyr-110). Inhibitors of Src family kinases, such as Src1, and the multi-kinase inhibitor bafetinib both suppressed PAR2-induced TRPV4 activation and associated mechanical hyperalgesia in animal models [73,85]. Another study proposed the participation of neutrophil elastase, a biased agonist of PAR2, and the activation of Rho-kinase signaling pathway in the sensitization of TRPV4 by PAR2 [61]. Further, it has been found that both cathepsin S and neutrophil elastase-biased agonism of PAR2 results in Gs-mediated cAMP formation, as well as the activation of adenylyl cyclase and PKA, which ultimately activate TRPV4 and sensitize nociceptive neurons to cause inflammatory pain [30,45]. PAR2-TRPV4 coupling is supported primarily by functional/signaling assays (indirect modulation) rather than direct molecular interactions. Distinguishing direct binding from second messenger modulation is critical, as it dictates selective therapeutic strategies (protein–protein interaction disruption versus pathway inhibition/biased ligands).

Most studies investigating PAR2–TRPV4 coupling derive from rodent models, in which PAR2 activation sensitizes TRPV4 to promote mechanical hyperalgesia. However, recent human DRG transcriptomic datasets including single-nucleus and spatial RNA sequencing, indicate that TRPV4 transcripts are expressed at low abundance or are rarely detected across human nociceptor subtypes [86,87], whereas TRPV1 and TRPA1 are robustly expressed. Thus, while direct neuronal PAR2–TRPV4 interactions are well supported in rodents, their presence in human DRG remains unverified and should be considered with appropriate translational caution. Given these species differences, clinical targeting of the PAR2–TRPV4 axis may be better directed toward non-neuronal compartments (e.g., keratinocytes), where TRPV4 is abundantly expressed in humans and can modulate nociceptor excitability via mediator release (e.g., endothelin-1) [88,89].

### 4.3. TRPA1

TRPA1, the only member of the ankyrin subfamily of TRP channels, is a non-selective cation channel expressed in primary sensory neurons, including subsets of DRG and TG neurons [83,90,91,92,93]. It functions as a chemosensor and is activated by a broad range of endogenous proalgesic molecules (e.g., nitro-oleic acid, bradykinin, H_2_O_2_) [94], and exogenous irritants including menthol, horseradish, mustard oil, allicin derived from garlic extract, cinnamaldehyde from cinnamon, and acrolein from exhaust fumes [12,95,96]. The functional interaction between PAR2 and TRPA1 is supported by five independent studies. Co-localization of the two receptors has been demonstrated in DRG neurons [62]. Functional interaction between PAR2 and TRPA1 has also been reported, explaining one of the mechanisms behind PAR2-mediated inflammatory pain [62,76,78]. Electrophysiological studies using TRPA1-transfected HEK293 cells and cultured DRG neurons showed that PAR2 activation significantly potentiated TRPA1 currents; see Figure 3C [62]. Moreover, this potentiation was suppressed with the application of PLC inhibitor or phosphatidylinositol-4,5-bisphosphate (PIP_2_), highlighting their role in TRPA1 sensitization following PAR2 activation; see Figure 2. In vivo studies showed that sub-inflammatory doses of PAR2 agonists (e.g., SL-NH_2_) enhanced nocifensive behaviors induced by TRPA1 agonists such as Allyl isothiocyanate (AITC) and cinnamaldehyde. Moreover, TRPA1 inhibition using the selective antagonist N-(4-tert-butylphenyl)-4-(3-chloropyridin-2-yl)piperazine-1-carboxamide (AP18) significantly reduced spinal Fos expression and referred hyperalgesia in a pancreatitis model, confirming TRPA1 as a downstream effector of PAR2 signaling [78]. A similar study also revealed that the activation of PAR2 and TRPA1 contributes to cystitis-associated pain, indicating that their inhibition might be a potential therapeutic approach [76].

### 4.4. ASIC3

Acid-sensing ion channel 3 (ASIC3) is a proton-gated, non-selective cation channel within the acid-sensing ion channel (ASIC) family, which comprises six subunits that assemble as trimeric complexes in mammals: ASIC1a, ASIC1b, ASIC2a, ASIC2b, ASIC3, and ASIC4 [97]. Most of the ASIC subunits are expressed in both DRG and primary sensory neurons and contribute to acidosis-evoked pain [98,99,100]. ASIC3, in particular, exhibits high sensitivity to changes in pH, with a pH_50_ near 6.5, and is predominantly expressed in DRG and primary afferent sensory neurons [98,100,101,102]. Physiological pH can decrease due to the extracellular release of protons during inflammation, ischemic stroke, and tissue injury, which in turn activates ASIC3 located at the sensory nerve terminals, evoking pain sensations [103,104]. Previous studies have shown that both ASIC3 and PAR2 play essential roles in the pathogenesis of inflammatory pain [54,101] and two studies provide evidence for a functional interaction between them, which contributes to acidosis-evoked nociception [63,80]. At the cellular level, it has been demonstrated that activation of PAR2 by PAR2-AP or trypsin potentiated ASIC3 currents in both DRG neurons and Chinese Hamster Ovary (CHO) cells co-transfected with ASIC3 and PAR2; see Figure 3D [63]. Moreover, the potentiation of ASIC3 currents by PAR2-AP was blocked by GDP-β-S (a G protein inhibitor), PLC, PKC, and PKA inhibitors, indicating the involvement of these kinases in the intracellular mechanisms underlying this potentiation [63]. In behavioral studies, it has been shown that intraplantar pretreatment with PAR2-AP enhanced acidosis-evoked nociceptive behaviors in a dose-dependent manner [63]. Additionally, in a model of esophageal inflammation (nonerosive reflux disease, NERD), PAR2 activation increased ATP release from human esophageal epithelial cells through a mechanism involving ASIC3 sensitization [80].

### 4.5. P2X3

P2X3 is a ligand-gated ion channel that belongs to the purinergic P2X receptor family and is uniquely expressed in nociceptive primary afferent neurons [81,91,105,106]. Among the seven known P2X subtypes, P2X3 is the most selectively expressed in sensory neurons and plays a critical role in mediating pain transmission in response to extracellular ATP, which is released following tissue injury or inflammation [81,91,105,106,107]. Evidence from three independent studies supports a functional interaction between PAR2 and P2X3. Co-expression of the two receptors in rat DRG neurons has been demonstrated [81], and electrophysiological recordings reveal that PAR2 activation, via SL-NH_2_ or trypsin, potentiates α,β-methylene-ATP–evoked P2X3 currents in DRG neurons; see Figure 3E. Moreover, the application of PKC and PKA inhibitors significantly suppressed this potentiation, while the application of their activators enhanced P2X3 current [64]. Conversely, a similar study revealed that PAR2 activation significantly decreases the amplitude of P2X3 currents, while simultaneously accelerating the opening of P2X3 channels [82]. Beyond changes in current amplitude, PAR2 also promotes P2X3 receptor trafficking to the plasma membrane, enhancing receptor availability. This process is accompanied by ERK phosphorylation in DRG neurons and correlates with increased nocifensive behaviors in vivo [64]; see Figure 2. In a behavioral assessment, it was demonstrated that a PAR2 agonist increased the nocifensive behavior induced by α,β-methylene-ATP, and the dose of the latter required to produce nociception was 100-fold lower in the inflamed tissue. Furthermore, the same study showed that the administration of PAR2 agonist also increased α,β-methylene-ATP-induced Fos expression in the dorsal horn neurons [81].

### 4.6. Cav3.2 and Kv7

Voltage-gated ion channels are essential mediators of membrane excitability contributing to neuronal signaling. Among these, Cav3.2 (T-type calcium channels) and Kv7 (M-current) have emerged as functionally relevant targets of PAR2 signaling in inflammatory pain [48,71]. Cav3.2 channels facilitate low-threshold calcium influx, leading to increased sensory neurons excitability. PAR2 activation in the bladder induced prostanoid-dependent referred hyperalgesia in a mouse model of bladder inflammation, with Cav3.2 channel contributing to its maintenance. Specifically, PAR2 activation enhanced cyclooxygenase-2 (COX-2) expression and PGE_2_ production, inducing an upregulated Cav3.2 expression through a PKA-dependent pathway. Pharmacological blockade of Cav3.2 reversed PAR2-induced hyperalgesia, highlighting its functional contribution to visceral pain amplification [71]. Conversely, Kv7 channels act as stabilizers of neuronal membrane potential by suppressing excitability through the M-current [48]. In DRG neurons, PAR2 activation inhibits Kv7-mediated M-type potassium currents through a PLC-dependent mechanism; see Figure 3F. This inhibition involves intracellular Ca^2+^ elevation and depletion of membrane PIP_2_, leading to membrane depolarization and increased neuronal firing. In vivo, administration of either a PAR2 agonist or a Kv7 channel blocker (XE991) enhanced nociceptive behaviors [48].

## 5. Conclusions

PAR2 is a GPCR activated by proteases released during inflammation, such as trypsin, tryptase, elastase, and cathepsin S. It is co-expressed with multiple nociceptive ion channels and plays a central role in inflammatory pain, mostly through modulation of their activity through diverse signaling pathways. To date, six ion channels, TRPV1, TRPV4, TRPA1, ASIC3, P2X3, Cav3.2 and Kv7 (M-current) channels, have been reported to interact with PAR2 leading to enhanced nociception during inflammation. These interactions are mediated via cellular mechanisms involving PLCβ, PKC, PKA, ERK, and membrane trafficking, and can arise from both canonical and biased signaling modes. PAR2-induced sensitization of these channels contributes to thermal, mechanical, chemical, and visceral hyperalgesia in various in vivo models. Given that PAR2 plays a role in maintaining normal physiological homeostasis, its direct antagonism may lead to adverse effects. Therefore, targeting the specific molecular interactions between PAR2 and these ion channels may represent a promising therapeutic strategy for the treatment of inflammatory pain. Future research should focus on defining the structural and contextual basis of this crosstalk to enable more precise intervention strategies.

## Data Availability

No new data were created or analyzed in this study. Data sharing is not applicable to this article.

## Abbreviations

The following abbreviations are used in this manuscript:

ATP	Adenosine triphosphate
ASIC3	Acid-sensing ion channel 3
cAMP	cyclic adenosine monophosphate
CGRP	Calcitonin gene-related peptide
DAG	Diacylglycerol
DRG	Dorsal root ganglia
ERK	Extracellular Signal-Regulated Kinase
Fos	an immediate early gene product used as a marker of neuronal activation
GPCR	G protein-coupled receptor
HEK	human embryonic kidney
IP_3_	Inositol Trisphosphate
Kv7	Voltage-gated potassium channels
MAPK	Mitogen-Activated Protein Kinase
PAR2	Protease-activated receptor 2
PAR2-AP	PAR2-activating peptide
PGE_2_	prostaglandin E_2_
PIP_2_	Phosphatidylinositol 4,5-bisphosphate
PKA	Protein kinase A
PKC	Protein kinase C
PLCβ	Phospholipase C-β
P2X3	P2X purinoceptor 3
SP	Substance P
SL-NH_2_	Ser-Leu amide (PAR2-agonist peptide)
SLIGRL-NH_2_	H-Ser-Leu-Ile-Gly-Arg-Leu-NH_2_ (PAR2-agonist peptide)
TG	Trigeminal ganglia
TL	Tethered ligand
TRPA1	Transient receptor potential ankyrin 1
TRPV1, TRPV4	Transient receptor potential vanilloid 1 and 4

## References

[1] Layne-Stuart C.M., Carpenter A.L. (2022). Chronic Pain Considerations in Patients with Cardiovascular Disease. Anesthesiol. Clin..

[2] Zhang Y.H., Adamo D., Liu H., Wang Q., Wu W., Zheng Y.L., Wang X.Q. (2023). Editorial: Inflammatory Pain: Mechanisms, Assessment, and Intervention. Front. Mol. Neurosci..

[3] Ji R.R., Xu Z.Z., Gao Y.J. (2014). Emerging Targets in Neuroinflammation-Driven Chronic Pain. Nat. Rev. Drug Discov..

[4] Muley M.M., Krustev E., McDougall J.J. (2016). Preclinical Assessment of Inflammatory Pain. CNS Neurosci. Ther..

[5] Simpson J.A., Fitch W. (1988). Chapter 5—Sensation. Applied Neurophysiology.

[6] Matsuda M., Huh Y., Ji R.-R. (2019). Roles of Inflammation, Neurogenic Inflammation, and Neuroinflammation in Pain. J. Anesth..

[7] Guan Z., Hellman J., Schumacher M. (2016). Contemporary Views on Inflammatory Pain Mechanisms: TRPing over Innate and Microglial Pathways. F1000Research.

[8] Prescott S.A., Ratté S. (2017). Somatosensation and Pain. Conn’s Translational Neuroscience.

[9] Julius D., Basbaum A.I. (2001). Molecular Mechanisms of Nociception. Nature.

[10] Ji R.R., Nackley A., Huh Y., Terrando N., Maixner W. (2018). Neuroinflammation and Central Sensitization in Chronic and Widespread Pain. Anesthesiology.

[11] Moore C., Gupta R., Jordt S.E., Chen Y., Liedtke W.B. (2018). Regulation of Pain and Itch by TRP Channels. Neurosci. Bull..

[12] Bautista D.M., Jordt S.E., Nikai T., Tsuruda P.R., Read A.J., Poblete J., Yamoah E.N., Basbaum A.I., Julius D. (2006). TRPA1 Mediates the Inflammatory Actions of Environmental Irritants and Proalgesic Agents. Cell.

[13] Kidd B.L., Urban L.A. (2001). Mechanisms of Inflammatory Pain. Br. J. Anaesth..

[14] Ji R.R., Gereau R.W., Malcangio M., Strichartz G.R. (2009). MAP Kinase and Pain. Brain Res. Rev..

[15] Obata K., Yamanaka H., Dai Y., Tachibana T., Fukuoka T., Tokunaga A., Yoshikawa H., Noguchi K. (2003). Differential Activation of Extracellular Signal-Regulated Protein Kinase in Primary Afferent Neurons Regulates Brain-Derived Neurotrophic Factor Expression after Peripheral Inflammation and Nerve Injury. J. Neurosci..

[16] Gold M.S., Levine J.D., Correa A.M. (1998). Modulation of TTX-R *I*_Na_ by PKC and PKA and Their Role in PGE_2_-Induced Sensitization of Rat Sensory Neurons In Vitro. J. Neurosci..

[17] Ji R.R., Samad T.A., Jin S.X., Schmoll R., Woolf C.J. (2002). p38 MAPK Activation by NGF in Primary Sensory Neurons after Inflammation Increases TRPV1 Levels and Maintains Heat Hyperalgesia. Neuron.

[18] Amaya F., Shimosato G., Nagano M., Ueda M., Hashimoto S., Tanaka Y., Suzuki H., Tanaka M. (2004). NGF and GDNF Differentially Regulate TRPV1 Expression That Contributes to Development of Inflammatory Thermal Hyperalgesia. Eur. J. Neurosci..

[19] Amaya F., Oh-Hashi K., Naruse Y., Iijima N., Ueda M., Shimosato G., Tominaga M., Tanaka Y., Tanaka M. (2003). Local Inflammation Increases Vanilloid Receptor 1 Expression within Distinct Subgroups of DRG Neurons. Brain Res..

[20] Latremoliere A., Woolf C.J. (2009). Central Sensitization: A Generator of Pain Hypersensitivity by Central Neural Plasticity. J. Pain.

[21] Moore K.A., Kohno T., Karchewski L.A., Scholz J., Baba H., Woolf C.J. (2002). Partial Peripheral Nerve Injury Promotes a Selective Loss of GABAergic Inhibition in the Superficial Dorsal Horn of the Spinal Cord. J. Neurosci..

[22] Fu K.Y., Light A.R., Maixner W. (2000). Relationship between Nociceptor Activity, Peripheral Edema, Spinal Microglial Activation and Long-Term Hyperalgesia Induced by Formalin. Neuroscience.

[23] Berta T., Park C.K., Xu Z.Z., Xie R.G., Liu T., Lü N., Liu Y.C., Ji R.R. (2014). Extracellular Caspase-6 Drives Murine Inflammatory Pain via Microglial TNF-α Secretion. J. Clin. Investig..

[24] Scholz J., Woolf C.J. (2007). The Neuropathic Pain Triad: Neurons, Immune Cells and Glia. Nat. Neurosci..

[25] Liu T., Gao Y.J., Ji R.R. (2012). Emerging Role of Toll-like Receptors in the Control of Pain and Itch. Neurosci. Bull..

[26] Raja S.N., Carr D.B., Cohen M., Finnerup N.B., Flor H., Gibson S., Keefe F.J., Mogil J.S., Ringkamp M., Sluka K.A. (2020). The Revised International Association for the Study of Pain Definition of Pain: Concepts, Challenges, and Compromises. Pain.

[27] Woolf C.J. (2011). Central Sensitization: Implications for the Diagnosis and Treatment of Pain. Pain.

[28] Schaible H.G. (2014). Nociceptive Neurons Detect Cytokines in Arthritis. Arthritis Res. Ther..

[29] Peach C.J., Edgington-Mitchell L.E., Bunnett N.W., Schmidt B.L. (2023). Protease-Activated Receptors in Health and Disease. Physiol. Rev..

[30] Zhao P., Lieu T., Barlow N., Metcalf M., Veldhuis N.A., Jensen D.D., Kocan M., Sostegni S., Haerteis S., Baraznenok V. (2014). Cathepsin S Causes Inflammatory Pain via Biased Agonism of PAR2 and TRPV4. J. Biol. Chem..

[31] Kume M., Ahmad A., Shiers S., Burton M.D., DeFea K.A., Vagner J., Dussor G., Boitano S., Price T.J. (2023). C781, a β-Arrestin Biased Antagonist at Protease-Activated Receptor-2 (PAR2), Displays in Vivo Efficacy Against Protease-Induced Pain in Mice. J. Pain.

[32] Gilman A.G. (1987). G Proteins: Transducers of Receptor-Generated Signals. Annu. Rev. Biochem..

[33] Nystedt S., Emilsson K., Wahlestedt C., Sundelin J. (1994). Molecular Cloning of a Potential Proteinase Activated Receptor. Proc. Natl. Acad. Sci. USA.

[34] Chandrabalan A., Ramachandran R. (2021). Molecular Mechanisms Regulating Proteinase-Activated Receptors (PARs). FEBS J..

[35] Heuberger D.M., Schuepbach R.A. (2019). Protease-Activated Receptors (PARs): Mechanisms of Action and Potential Therapeutic Modulators in PAR-Driven Inflammatory Diseases. Thromb. J..

[36] Zhao P., Metcalf M., Bunnett N.W. (2014). Biased Signaling of Protease-Activated Receptors. Front. Endocrinol..

[37] Macfarlane S.R., Sloss C.M., Cameron P., Kanke T., McKenzie R.C., Plevin R. (2005). The Role of Intracellular Ca^2+^ in the Regulation of Proteinase-Activated Receptor-2 Mediated Nuclear Factor Kappa B Signalling in Keratinocytes. Br. J. Pharmacol..

[38] DeFea K.A., Zalevsky J., Thoma M.S., Dery O., Mullins R.D., Bunnett N.W. (2000). Beta-Arrestin-Dependent Endocytosis of Proteinase-Activated Receptor 2 Is Required for Intracellular Targeting of Activated ERK1/2. J. Cell Biol..

[39] Déry O., Thoma M.S., Wong H., Grady E.F., Bunnett N.W. (1999). Trafficking of Proteinase-Activated Receptor-2 and Beta-Arrestin-1 Tagged with Green Fluorescent Protein. Beta-Arrestin-Dependent Endocytosis of a Proteinase Receptor. J. Biol. Chem..

[40] Jacob C., Cottrell G.S., Gehringer D., Schmidlin F., Grady E.F., Bunnett N.W. (2005). C-Cbl Mediates Ubiquitination, Degradation, and down-Regulation of Human Protease-Activated Receptor 2. J. Biol. Chem..

[41] Hasdemir B., Murphy J.E., Cottrell G.S., Bunnett N.W. (2009). Endosomal Deubiquitinating Enzymes Control Ubiquitination and Down-Regulation of Protease-Activated Receptor 2. J. Biol. Chem..

[42] Böhm S.K., Kong W., Brömme D., Smeekens S.P., Anderson D.C., Connolly A., Kahn M., Nelken N.A., Coughlin S.R., Payan D.G. (1996). Molecular Cloning, Expression and Potential Functions of the Human Proteinase-Activated Receptor-2. Biochem. J..

[43] Hollenberg M.D., Mihara K., Polley D., Suen J.Y., Han A., Fairlie D.P., Ramachandran R. (2014). Biased Signalling and Proteinase-Activated Receptors (PARs): Targeting Inflammatory Disease. Br. J. Pharmacol..

[44] Kelso E.B., Lockhart J.C., Hembrough T., Dunning L., Plevin R., Hollenberg M.D., Sommerhoff C.P., McLean J.S., Ferrell W.R. (2006). Therapeutic Promise of Proteinase-Activated Receptor-2 Antagonism in Joint Inflammation. J. Pharmacol. Exp. Ther..

[45] Zhao P., Lieu T.M., Barlow N., Sostegni S., Haerteis S., Korbmacher C., Liedtke W., Jimenez-Vargas N.N., Vanner S.J., Bunnett N.W. (2015). Neutrophil Elastase Activates Protease-Activated Receptor-2 (PAR2) and Transient Receptor Potential Vanilloid 4 (TRPV4) to Cause Inflammation and Pain. J. Biol. Chem..

[46] Rothmeier A.S., Ruf W. (2011). Protease-Activated Receptor 2 Signaling in Inflammation. Semin. Immunopathol..

[47] Cheng L., Xia F., Li Z., Shen C., Yang Z., Hou H., Sun S., Feng Y., Yong X., Tian X. (2023). Structure, Function and Drug Discovery of GPCR Signaling. Mol. Biomed..

[48] Linley J.E., Rose K., Patil M., Robertson B., Akopian A.N., Gamper N. (2008). Inhibition of M Current in Sensory Neurons by Exogenous Proteases: A Signaling Pathway Mediating Inflammatory Nociception. J. Neurosci..

[49] Chen Y., Yang C., Wang Z.J. (2011). Proteinase-Activated Receptor 2 Sensitizes Transient Receptor Potential Vanilloid 1, Transient Receptor Potential Vanilloid 4, and Transient Receptor Potential Ankyrin 1 in Paclitaxel-Induced Neuropathic Pain. Neuroscience.

[50] Muley M.M., Reid A.R., Botz B., Bölcskei K., Helyes Z., McDougall J.J. (2016). Neutrophil Elastase Induces Inflammation and Pain in Mouse Knee Joints via Activation of Proteinase-Activated Receptor-2. Br. J. Pharmacol..

[51] Lopes A.H., Brandolini L., Aramini A., Bianchini G., Silva R.L., Zaperlon A.C., Verri W.A., Alves-Filho J.C., Cunha F.Q., Teixeira M.M. (2016). DF2755A, a Novel Non-Competitive Allosteric Inhibitor of CXCR1/2, Reduces Inflammatory and Post-Operative Pain. Pharmacol. Res..

[52] Cunha T.M., Barsante M.M., Guerrero A.T., Verri W.A., Ferreira S.H., Coelho F.M., Bertini R., Di Giacinto C., Allegretti M., Cunha F.Q. (2008). Treatment with DF 2162, a Non-Competitive Allosteric Inhibitor of CXCR1/2, Diminishes Neutrophil Influx and Inflammatory Hypernociception in Mice. Br. J. Pharmacol..

[53] Buddenkotte J., Stroh C., Engels I.H., Moormann C., Shpacovitch V.M., Seeliger S., Vergnolle N., Vestweber D., Luger T.A., Schulze-Osthoff K. (2005). Agonists of Proteinase-Activated Receptor-2 Stimulate Upregulation of Intercellular Cell Adhesion Molecule-1 in Primary Human Keratinocytes via Activation of NF-Kappa B. J. Investig. Dermatol..

[54] Steinhoff M., Vergnolle N., Young S.H., Tognetto M., Amadesi S., Ennes H.S., Trevisani M., Hollenberg M.D., Wallace J.L., Caughey G.H. (2000). Agonists of Proteinase-Activated Receptor 2 Induce Inflammation by a Neurogenic Mechanism. Nat. Med..

[55] Kong W., McConalogue K., Khitin L.M., Hollenberg M.D., Payan D.G., Böhm S.K., Bunnett N.W. (1997). Luminal Trypsin May Regulate Enterocytes through Proteinase-Activated Receptor 2. Proc. Natl. Acad. Sci. USA.

[56] Liu Q., Weng H.J., Patel K.N., Tang Z., Bai H., Steinhoff M., Dong X. (2011). The Distinct Roles of Two GPCRs, MrgprC11 and PAR2, in Itch and Hyperalgesia. Sci. Signal..

[57] Hassler S.N., Kume M., Mwirigi J.M., Ahmad A., Shiers S., Wangzhou A., Ray P.R., Belugin S.N., Naik D.K., Burton M.D. (2020). The Cellular Basis of Protease-Activated Receptor 2-Evoked Mechanical and Affective Pain. JCI Insight.

[58] Lieu T., Savage E., Zhao P., Edgington-Mitchell L., Barlow N., Bron R., Poole D.P., McLean P., Lohman R.J., Fairlie D.P. (2016). Antagonism of the Proinflammatory and Pronociceptive Actions of Canonical and Biased Agonists of Protease-Activated Receptor-2. Br. J. Pharmacol..

[59] Mrozkova P., Spicarova D., Palecek J. (2021). Spinal PAR2 Activation Contributes to Hypersensitivity Induced by Peripheral Inflammation in Rats. Int. J. Mol. Sci..

[60] Dai Y., Moriyama T., Higashi T., Togashi K., Kobayashi K., Yamanaka H., Tominaga M., Noguchi K. (2004). Proteinase-Activated Receptor 2-Mediated Potentiation of Transient Receptor Potential Vanilloid Subfamily 1 Activity Reveals a Mechanism for Proteinase-Induced Inflammatory Pain. J. Neurosci..

[61] Sostegni S., Diakov A., McIntyre P., Bunnett N., Korbmacher C., Haerteis S. (2015). Sensitisation of TRPV4 by PAR2 Is Independent of Intracellular Calcium Signalling and Can Be Mediated by the Biased Agonist Neutrophil Elastase. Pflügers Arch.-Eur. J. Physiol..

[62] Dai Y., Wang S., Tominaga M., Yamamoto S., Fukuoka T., Higashi T., Kobayashi K., Obata K., Yamanaka H., Noguchi K. (2007). Sensitization of TRPA1 by PAR2 Contributes to the Sensation of Inflammatory Pain. J. Clin. Investig..

[63] Wu J., Liu T.T., Zhou Y.M., Qiu C.Y., Ren P., Jiao M., Hu W.P. (2017). Sensitization of ASIC3 by Proteinase-Activated Receptor 2 Signaling Contributes to Acidosis-Induced Nociception. J. Neuroinflamm..

[64] Wang S., Dai Y., Kobayashi K., Zhu W., Kogure Y., Yamanaka H., Wan Y., Zhang W., Noguchi K. (2012). Potentiation of the P2X3 ATP Receptor by PAR-2 in Rat Dorsal Root Ganglia Neurons, through Protein Kinase-Dependent Mechanisms, Contributes to Inflammatory Pain. Eur. J. Neurosci..

[65] Amadesi S., Nie J., Vergnolle N., Cottrell G.S., Grady E.F., Trevisani M., Manni C., Geppetti P., McRoberts J.A., Ennes H. (2004). Protease-Activated Receptor 2 Sensitizes the Capsaicin Receptor Transient Receptor Potential Vanilloid Receptor 1 to Induce Hyperalgesia. J. Neurosci. Off. J. Soc. Neurosci..

[66] Amadesi S., Cottrell G.S., Divino L., Chapman K., Grady E.F., Bautista F., Karanjia R., Barajas-Lopez C., Vanner S., Vergnolle N. (2006). Protease-Activated Receptor 2 Sensitizes TRPV1 by Protein Kinase Cε- and A-Dependent Mechanisms in Rats and Mice. J. Physiol..

[67] Helyes Z., Sándor K., Borbély E., Tékus V., Pintér E., Elekes K., Tóth D.M., Szolcsányi J., McDougall J.J. (2010). Involvement of Transient Receptor Potential Vanilloid 1 Receptors in Protease-Activated Receptor-2-Induced Joint Inflammation and Nociception. Eur. J. Pain.

[68] Russell F., Schuelert N., Veldhoen V., Hollenberg M., McDougall J. (2012). Activation of PAR2 Receptors Sensitizes Primary Afferents and Causes Leukocyte Rolling and Adherence in the Rat Knee Joint. Br. J. Pharmacol..

[69] Mrozkova P., Spicarova D., Palecek J. (2016). Hypersensitivity Induced by Activation of Spinal Cord PAR2 Receptors Is Partially Mediated by TRPV1 Receptors. PLoS ONE.

[70] Zhong B., Ma S., Wang D.H. (2019). Protease-Activated Receptor 2 Protects against Myocardial Ischemia-Reperfusion Injury through the Lipoxygenase Pathway and TRPV1 Channels. Exp. Ther. Med..

[71] Tsubota M., Ozaki T., Hayashi Y., Okawa Y., Fujimura A., Sekiguchi F., Nishikawa H., Kawabata A. (2018). Prostanoid-Dependent Bladder Pain Caused by Proteinase-Activated Receptor-2 Activation in Mice: Involvement of TRPV1 and T-Type Ca^2+^ Channels. J. Pharmacol. Sci..

[72] Grant A.D., Cottrell G.S., Amadesi S., Trevisani M., Nicoletti P., Materazzi S., Altier C., Cenac N., Zamponi G.W., Bautista-Cruz F. (2007). Protease-Activated Receptor 2 Sensitizes the Transient Receptor Potential Vanilloid 4 Ion Channel to Cause Mechanical Hyperalgesia in Mice. J. Physiol..

[73] Poole D.P., Amadesi S., Veldhuis N.A., Abogadie F.C., Lieu T.M., Darby W., Liedtke W., Lew M.J., McIntyre P., Bunnett N.W. (2013). Protease-Activated Receptor 2 (PAR2) Protein and Transient Receptor Potential Vanilloid 4 (TRPV4) Protein Coupling Is Required for Sustained Inflammatory Signaling. J. Biol. Chem..

[74] Grace M.S., Lieu T., Darby B., Abogadie F.C., Veldhuis N., Bunnett N.W., Mcintyre P. (2014). The Tyrosine Kinase Inhibitor Bafetinib Inhibits PAR2-Induced Activation of TRPV4 Channels In Vitro and Pain In Vivo. Br. J. Pharmacol..

[75] Rayees S., Joshi J.C., Tauseef M., Anwar M., Baweja S., Rochford I., Joshi B., Hollenberg M.D., Reddy S.P., Mehta D. (2019). PAR2-Mediated cAMP Generation Suppresses TRPV4-Dependent Ca^2+^ Signaling in Alveolar Macrophages to Resolve TLR4-Induced Inflammation. Cell Rep..

[76] Chen D., Liu N., Li M., Liang S. (2016). Blocking PAR2 Alleviates Bladder Pain and Hyperactivity via TRPA1 Signal. Transl. Neurosci..

[77] Kopruszinski C.M., Linley J.E., Thornton P., Walker A.S., Newton P., Podichetty S., Ruparel R.H., Moreira de Souza L.H., Navratilova E., Meno-Tetang G. (2025). Efficacy of MEDI0618, a pH-Dependent Monoclonal Antibody Targeting PAR2, in Preclinical Models of Migraine. Brain.

[78] Terada Y., Fujimura M., Nishimura S., Tsubota M., Sekiguchi F., Nishikawa H., Kawabata A. (2013). Contribution of TRPA1 as a Downstream Signal of Proteinase-Activated Receptor-2 to Pancreatic Pain. J. Pharmacol. Sci..

[79] Ito M., Ono K., Hitomi S., Nodai T., Sago T., Yamaguchi K., Harano N., Gunnjigake K., Hosokawa R., Kawamoto T. (2017). Prostanoid-Dependent Spontaneous Pain and PAR2-Dependent Mechanical Allodynia Following Oral Mucosal Trauma: Involvement of TRPV1, TRPA1 and TRPV4. Mol. Pain.

[80] Wu L., Oshima T., Shan J., Sei H., Tomita T., Ohda Y., Fukui H., Watari J., Miwa H. (2015). PAR-2 Activation Enhances Weak Acid-Induced ATP Release through TRPV1 and ASIC Sensitization in Human Esophageal Epithelial Cells. Am. J. Physiol. Liver Physiol..

[81] Zhu W.J., Dai Y., Fukuoka T., Yamanaka H., Kobayashi K., Obata K., Wang S., Noguchi K. (2006). Agonist of Proteinase-Activated Receptor 2 Increases Painful Behavior Produced by Alpha, Beta-Methylene Adenosine 5′-Triphosphate. NeuroReport.

[82] Lu Z.J., Miao X.R., Wu J.X., Wang X.Y., Miao Q., Yu W.F. (2010). Acute PAR2 Activation Reduces α, β-MeATP Sensitive Currents in Rat Dorsal Root Ganglion Neurons. NeuroReport.

[83] Samanta A., Hughes T.E.T., Moiseenkova-Bell V.Y. (2018). Transient Receptor Potential (TRP) Channels. Membrane Protein Complexes: Structure and Function.

[84] Alier K.A., Endicott J.A., Stemkowski P.L., Cenac N., Cellars L., Chapman K., Andrade-Gordon P., Vergnolle N., Smith P.A. (2008). Intrathecal Administration of Proteinase-Activated Receptor-2 Agonists Produces Hyperalgesia by Exciting the Cell Bodies of Primary Sensory Neurons. J. Pharmacol. Exp. Ther..

[85] Wegierski T., Lewandrowski U., Müller B., Sickmann A., Walz G. (2009). Tyrosine Phosphorylation Modulates the Activity of TRPV4 in Response to Defined Stimuli. J. Biol. Chem..

[86] Nguyen M.Q., von Buchholtz L.J., Reker A.N., Ryba N.J., Davidson S. (2021). Single-Nucleus Transcriptomic Analysis of Human Dorsal Root Ganglion Neurons. Elife.

[87] Tavares-Ferreira D., Shiers S., Ray P.R., Wangzhou A., Jeevakumar V., Sankaranarayanan I., Cervantes A.M., Reese J.C., Chamessian A., Copits B.A. (2022). Spatial Transcriptomics of Dorsal Root Ganglia Identifies Molecular Signatures of Human Nociceptors. Sci. Transl. Med..

[88] Moore C., Cevikbas F., Pasolli H.A., Chen Y., Kong W., Kempkes C., Parekh P., Lee S.H., Kontchou N.-A., Yeh I. (2013). UVB Radiation Generates Sunburn Pain and Affects Skin by Activating Epidermal TRPV4 Ion Channels and Triggering Endothelin-1 Signaling. Proc. Natl. Acad. Sci. USA.

[89] Chen Y., Fang Q., Wang Z., Zhang J.Y., MacLeod A.S., Hall R.P., Liedtke W.B. (2016). Transient Receptor Potential Vanilloid 4 Ion Channel Functions as a Pruriceptor in Epidermal Keratinocytes to Evoke Histaminergic Itch. J. Biol. Chem..

[90] Nagata K., Duggan A., Kumar G., García-Añoveros J. (2005). Nociceptor and Hair Cell Transducer Properties of TRPA1, a Channel for Pain and Hearing. J. Neurosci. Off. J. Soc. Neurosci..

[91] Kobayashi K., Fukuoka T., Yamanaka H., Dai Y., Obata K., Tokunaga A., Noguchi K. (2005). Differential Expression Patterns of mRNAs for P2X Receptor Subunits in Neurochemically Characterized Dorsal Root Ganglion Neurons in the Rat. J. Comp. Neurol..

[92] Zygmunt P.M., Högestätt E.D. (2014). TRPA1. Mammalian Transient Receptor Potential (TRP) Cation Channels.

[93] Jordt S.E., Bautista D.M., Chuang H.H., McKemy D.D., Zygmunt P.M., Högestätt E.D., Meng I.D., Julius D. (2004). Mustard Oils and Cannabinoids Excite Sensory Nerve Fibres through the TRP Channel ANKTM1. Nature.

[94] Bandell M., Story G.M., Hwang S.W., Viswanath V., Eid S.R., Petrus M.J., Earley T.J., Patapoutian A. (2004). Noxious Cold Ion Channel TRPA1 Is Activated by Pungent Compounds and Bradykinin. Neuron.

[95] Jaquemar D., Schenker T., Trueb B. (1999). An Ankyrin-like Protein with Transmembrane Domains Is Specifically Lost after Oncogenic Transformation of Human Fibroblasts. J. Biol. Chem..

[96] Story G.M., Peier A.M., Reeve A.J., Eid S.R., Mosbacher J., Hricik T.R., Earley T.J., Hergarden A.C., Andersson D.A., Hwang S.W. (2003). ANKTM1, a TRP-like Channel Expressed in Nociceptive Neurons, Is Activated by Cold Temperatures. Cell.

[97] Wemmie J.A., Price M.P., Welsh M.J. (2006). Acid-Sensing Ion Channels: Advances, Questions and Therapeutic Opportunities. Trends Neurosci..

[98] Deval E., Lingueglia E. (2015). Acid-Sensing Ion Channels and Nociception in the Peripheral and Central Nervous Systems. Neuropharmacology.

[99] Price M.P., McIlwrath S.L., Xie J., Cheng C., Qiao J., Tarr D.E., Sluka K.A., Brennan T.J., Lewin G.R., Welsh M.J. (2001). The DRASIC Cation Channel Contributes to the Detection of Cutaneous Touch and Acid Stimuli in Mice. Neuron.

[100] Deval E., Noël J., Lay N., Alloui A., Diochot S., Friend V., Jodar M., Lazdunski M., Lingueglia E. (2008). ASIC3, a Sensor of Acidic and Primary Inflammatory Pain. EMBO J..

[101] Wemmie J.A., Taugher R.J., Kreple C.J. (2013). Acid-Sensing Ion Channels in Pain and Disease. Nat. Rev. Neurosci..

[102] Heusser S.A., Pless S.A. (2021). Acid-Sensing Ion Channels as Potential Therapeutic Targets. Trends Pharmacol. Sci..

[103] Steen K.H., Reeh P.W., Anton F., Handwerker H.O. (1992). Protons Selectively Induce Lasting Excitation and Sensitization to Mechanical Stimulation of Nociceptors in Rat Skin, In Vitro. J. Neurosci. Off. J. Soc. Neurosci..

[104] Basbaum A.I., Bautista D.M., Scherrer G., Julius D. (2009). Cellular and Molecular Mechanisms of Pain. Cell.

[105] Burnstock G. (2000). P2X Receptors in Sensory Neurones. Br. J. Anaesth..

[106] Dunn P.M., Zhong Y., Burnstock G. (2001). P2X Receptors in Peripheral Neurons. Prog. Neurobiol..

[107] Chizh B., Illés P. (2001). P2X Receptors and Nociception. Pharmacol. Rev..

